# An evidence-based and risk-adapted GSF versus GSF plus plerixafor mobilization strategy to obtain a sufficient CD34^+^ cell yield in the harvest for autologous stem cell transplants

**DOI:** 10.1016/j.tranon.2023.101811

**Published:** 2023-10-31

**Authors:** Milena Todorović Balint, Nikola Lemajić, Vladimir Jurišić, Sofija Pantelić, Dejana Stanisavljević, Nada Kraguljac Kurtović, Bela Balint

**Affiliations:** a Clinic for Hematology, University Clinical Center of Serbia, Belgrade, Serbia; b Medical Faculty, University of Belgrade, Serbia; c Faculty of Medical Sciences, University of Kragujevac, Serbia; d Institute for Oncology and Radiology of Serbia, Belgrade, Serbia; e Institute for Medical Statistics and Informatics, Belgrade, Serbia; f Department of Medical Sciences, Serbian Academy of Sciences and Arts, Serbia; g Faculty of Medicine of the Military Medical Academy, University of Defense, Belgrade, Serbia

**Keywords:** CD34^+^ cell mobilization, Cytokines, G-CSF, Plerixafor, Autologous transplantation

## Abstract

**Background:**

Plerixafor is a bicyclam molecule with the ability to reversibly bind to receptor CXCR-4 thus leading to an increased release of stem cells (SC) into the circulation. This study aims to evaluate the efficacy of G-CSF plus plerixafor versus G-CSF alone mobilizing regimens on the basis of CD34^+^ cell yield and engraftment kinetics following hematopoietic SC transplants.

**Methods:**

The study incorporated 173 patients with plasma cell neoplasms (PCN), Hodgkin's lymphoma (HL) and non-Hodgkin's lymphoma (NHL), undergoing mobilization and following autologous SC-transplant. For patients with mobilization failure and those predicted to be at risk of harvesting inadequate CD34^+^ yields (poor-responders), plerixafor was administered. Data was collected and compared in relation to the harvesting protocols used, cell quantification, cell-engraftment potential and overall clinical outcome.

**Results:**

A total of 101 patients received plerixafor (58.4 %) and the median CD34^+^increase was 312 %. Chemotherapy-mobilized PCN-patients required less plerixafor administration (*p* = 0.01), no difference was observed in lymphoma groups (*p* = 0.46). The median CD34^+^cell yield was 7.8 × 10^6^/kg bm. Patients requiring plerixafor achieved lower, but still comparable cell yields. Total cell dose infused was in correlation with engraftment kinetics. Patients requiring plerixafor had delayed platelet engraftment (*p* = 0.029).

**Conclusions:**

Adequately selected plerixafor administration reduces "mobilization-related-failure" rate and assure a high-level cell dose for SC transplants, with superior "therapeutic-potential" and safety profile. The mobilization strategy that incorporates "*just-in-time*" plerixafor administration, also leads to a reduction of hospitalization days and healthcare resource utilization. For definitive conclusions, further controlled/larger clinical trials concerning correlation of CD34^+^ cell count/yield, with hematopoietic reconstitution are required.

## Highlights

• The addition of plerixafor to cytokine G-CSF provide sufficient stem cell yield in harvest in poor mobilizers.
• Combined mobilization approach fulfills criteria of optimal approach for autologous stem cell transplantation, overcoming poor mobilization potential.
• The use of risk adapted mobilization strategies leads to a reduction of possible hospitalization days and healthcare resource utilization.

## Graphical abstract

**Figure:**
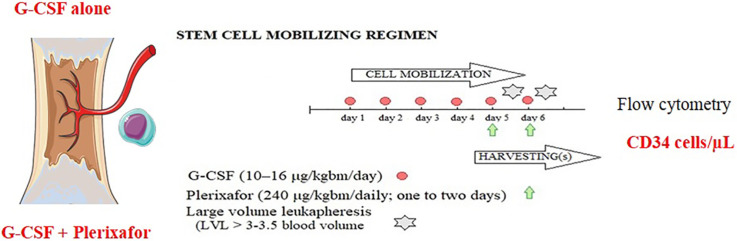

## Introduction

Autologous stem cell transplantation (ASCT) is a cornerstone in the treatment of plasma cell neoplasms (PCN), and relapsed non-Hodgkins's lymphoma (NHL) or Hodgkins's lymphoma (HL). According to the European Group for Blood & Marrow Transplantation (EMBT) registry data in Europe in 2021 more than 27 000 ASCT were performed [1]. While the absolute majority of hematopoietic stem cells (SC) reside in the bone marrow, due to patient comfort and favorable engraftment kinetics, mobilized circulating stem cells from the bloodstream are predominantly used. In the native state their numbers are inadequate to fulfill the requirements needed to successfully perform an ASCT. Their amount must be increased using pharmacological stimulation which leads to a 10 to a 100-fold increase [2]. There are various approaches to mobilize adequate numbers of CD34^+^ cells. The simplest form is mobilization termed steady state using cytokines only, G-CSF to be specific. Multiple doses of G-CSF induce a state of accelerated myeloid hematopoiesis increasing the total white blood cell (WBC) count and indirectly increasing the number of hematopoietic stem cells (HSC). In addition, G-CSF can disrupt key interactions, most notably chemokine receptor type 4/12 (CXCR4/CXCL12) signaling. Other pathways are also activated that may augment HSC mobilization by modulating CXCR4 signaling including complement and urokinase plasminogen activator (uPAR) activation [3,4]. It is well tolerated, does not require hospitalization, but it usually leads to a suboptimal HSC yield. Cytokine only mobilization can be further optimized by using chemotherapy, either disease specific chemotherapy or separate chemo-mobilization often using cyclophosphamide or etoposide [5]. The addition of chemotherapy leads to an increased HSC yield also while providing antitumor activity [6,7]. Despite the aforementioned benefits it often requires patient hospitalization and is associated with lower predictability of apheresis timing and higher rates of complications.

Plerixafor (AMD3100) is a bicyclam molecule and its mechanism of action is the reversible blockade of C-X-C chemokine receptor type 4 (CXCR-4), thereby inhibiting binding with stromal-cell derived factor 1 (SDF-1) [2]. Originally envisioned as a potential anti-HIV agent the drug soon found another use after showing high efficacy in increasing the number of white blood cells (WBC). The blockade of SDF-1*/*CXCR4 interaction results in the increased release of immature CD34^+^ hematopoietic stem cells in the circulation [2,8]. Shortly afterwards, the potential for peripheral blood stem cell (PBSC) mobilization with plerixafor and long-term engraftment was successfully demonstrated both in murine models and humans [9]. Since its approval, plerixafor has become a staple in the setting of high dose chemotherapy and ASCT [10]. Plerixafor can be used both in the steady state setting and in the setting of chemotherapy-based mobilization, but it is only registered for use in patients who are poor mobilizers [5].

The goal of mobilization is to ensure a minimum yield of ≥ 2 × 10^6^ CD34^+^ cells per kg of body mass (kg bm). Transplantations with a CD34^+^ cell dose ≥ 5 × 10^6^/kg bm are associated with faster engraftment and fewer complications. Despite all the advances in mobilization protocols, the failure rate varies between 6 % and 23 % [11]. Subsequently, the patients are unable to receive adequate treatment which leads to inferior outcomes [12], [13], [14].

The objective of this retrospective study is to evaluate the impact of plerixafor administration compared to the use of G-CSF alone on the basis of the apheresis protocol collection efficacy, the targeted cell yield and harvest quality, as well as the engraftment kinetics in patients with PCN and lymphomas. The study was conducted according to ethical principles of the Declaration of Helsinki, with Ethical board decision No 1322/XII-2.

## Materials and methods

A retrospective comparative study has been carried out involving 173 consecutive patients with PCN, NHL and HL undergoing HSC mobilization and subsequent ASCT between July 2019 and July 2022. All diagnoses were in concordance with the WHO Classification of Tumours of Haematopoietic and Lymphoid Tissues [15]. Disease staging was done by using the Ann Arbor system for lymphoma patients, and International Staging System (ISS) and Revised ISS (R-ISS) for Myeloma patients [16], [17], [18], [19]. FISH analysis was interpreted using m-SMART criteria [20]. Steady state mobilization was started with filgrastim (Zarzio®) using a dose of 10–16 μg/kg bm for four days regardless of leukocyte and CD34^+^ counts. On the fifth day of mobilization, the number of circulating CD34^+^ cells were analyzed using flow cytometry (BD FACSCanto II™). Chemo-mobilization was used for high-risk patients, those with suboptimal response to previous therapy, MM patients that were planned for tandem transplantation and finally patients that were expected to be poor mobilisers. For MM patients, disease specific mobilization CAD (cyclophosphamide, doxorubicin, dexamethasone) chemotherapy was used [21]. Lymphoma patients were mobilized after disease specific salvage chemotherapy regimens, containing platin or carboplatin ((*R*)-DHAP, (*R*)-ESHAP, (*R*)-ICE) [22,23]. In the case of achieving > 20 CD34^+^ cell/µL of peripheral blood (PB), the apheresis initiated, per international guidelines. When the number of cells was between 10 and 20 an individual approach was made based on the patient's overall status and treatment history. If the number of CD34^+^ cells was 〈 10/µL of PB, filgrastim dose was escalated and plerixafor was given to every patient. To reduce the need for multiple apheresis session patients, plerixafor was used even if the number of circulating cells was 〉 20 in the case of high WBC counts resulting in a low CD34^+^ SC to mononuclear cell ratio, and if the MM patient was planned for tandem transplantation with a need for ≥ 8 × 10^6^ CD34^+^cells/kg bm harvested. Plerixafor 240 μg/kg bm subcutaneously, was given 6 h before cell enumeration which ensured that the PBSC peak was achieved during apheresis. Patients were defined as predicted poor mobilisers or proven poor mobilisers per The Italian Group for Stem Cell Transplantation GITMO (Gruppo Italiano Trapianto di Midollo Osseo) guidelines [24]. In the case of inadequate yield < 2 × 10^6^ CD34^+^ cells/kg bm for lymphoma patients and single ASCT patients, or < 8 × 10^6^ CD34^+^ cells/kg bm for tandem ASCT, additional apheresis procedures were performed until the target values were reached. Optimal PBSC harvest was categorized as achieving ≥ 5 × 10^6^ CD34^+^ cells/kg bm for single transplantation and ≥ 10×10^6^ CD34^+^ cells/kg bm for tandem transplantation, adequate yield was categorized as achieving a yield of 2–5 × 10^6^ CD34^+^ cells/kg bm for single transplantation and 8–10×10^6^ CD34^+^ cells/kg bm for tandem transplantation. Inadequate harvest was defined as achieving a yield < 2 × 10^6^ CD34^+^ cells/kg bm and was considered as a mobilization failure [25], [26], [27]. Peripheral blood cells were collected using a Spectra Optia® apheresis system through a central venous catheter or through a peripheral venous line. The cells were cryopreserved at −140 °C. Dimethyl sulfoxide (DMSO) 99.9 % was used as a cryoprotective agent, in the final concentration of 8–10 %. Afterwards, the cell viability was assessed by trypan blue staining and by flow cytometry using 7-Amino- actinomycin (7-AAD) staining.

For Myeloma patients, high dose Melphalan (HD-Mel) 200 mg/m^2^ was the conditioning regimen of choice while for some patients, with regard to comorbidities, the dose was reduced to 140 mg/m^2^. Lymphoma patients were conditioned using a conventional BEAM regimen [28]. After conditioning the thawed autologous SC were infused. Filgrastim was started on day +5 after transplant, and was administered once daily to facilitate engraftment. Engraftment was defined as the first of 3 consecutive days with an absolute neutrophil count ≥ 0.5 × 10^9^/L (sustained ≥ 20×10^9^/L platelets and hemoglobin > 80 g/L, free of transfusion requirements) [29]. Treatment response was evaluated in accordance with the RECIL criteria for lymphomas, IMWG criteria for MM. For Plasmacytoma recommendations made by a European Expert Panel were used [30], [31], [32].

### Statistical analysis

All statistical analyses were performed using SPSS 21.0. A p value of 0.05 or less was chosen to define statistical significance. Descriptive statistical analyses were carried out on the study sample. Values were reported as proportions and percentages for categorical variables and as means and standard deviations or modes with ranges for continuous variables. Response to therapy before and after transplantation was evaluated using McNemar's test for matched pairs. For continuous data, if it was normally distributed, the statistical significance was assessed using the *t*-test. If the data were not normally distributed, the Mann-Whitney U test was used instead. Comparisons between proportions were made using the chi-square test.

## Results

### Patient characteristics

A total of 173 patients were hospitalized for stem cell mobilization between July 2019 and July 2022. Of these, 103 were male (59.5 %) while women comprised 70 patients (40.5 %). The median age at mobilization and apheresis was 54 years (range 20–69 years). PCN were most common diagnosis with 123 patients (71.1 %), with the leading diagnosis being MM, with 111 patients (64.1 %). NHL was more common than HL with 32 patients (18.5 %) compared to 18 patients (10.4 %). More than half of MM patients were R-ISS-2 (57.7 %). Half of the HL patients were stage IV, and 26 (83.8 %) NHL patients were stage IV. A quarter of the patients had received radiation therapy. The majority of PCN patients received one therapy line before transplantation, contrary the majority of lymphoma patients received two or more lines. Chemotherapy mobilization was carried out in 98 (56.6 %) of patients, while mobilization with G-CSF only was carried out in 75 (43.3 %) of patients. Patient characteristics are summarized in Table 1.

**Table 1:** Patient characteristics.

Variable	All patients
Age at diagnosis (min–max)	52 (17–68)
Age at mobilization median (min–max)	54 (20–69)
Age at transplantation(min–max)	54 (22–69)
Female N (%)	
Male N (%)	70 (40.5 %)
103 (59.5 %)	
Diagnosis	**N (%)**
PCN	123 (71.1 %)
MM	111 (64.1 %)
NHL	32 (18.5 %)
HL	18 (10.4 %)
MM ISS	
ISS1	37 (33.3 %)
ISS2	31 (27.9 %)
ISS3	40 (36 %)
N/A	3 (2.7 %)
MM R-ISS	
RISS1	23 (20.7 %)
RISS2	63 (56.7 %)
RISS3	13 (11.7 %)
N/A	12 (10.8 %)
HL stage	
Stage 1	0 (0 %)
Stage 2	8 (44.4 %)
Stage 3	1 (5.6 %)
Stage 4	9 (50 %)
NHL stage	
Stage 1	0 (0 %)
Stage 2	5 (15.6 %)
Stage 3	1 (3.1 %)
Stage 4	26 (81.2 %)
Radiotherapy	46 (26.6 %)
G-CSF only	72 (41.6 %)
G-CFF+ P	101 (58.4 %)
CAD	69 (39.9 %)
Salvage	29 (16.8 %)
Steady state	75 (43.3 %)
Tandem transplantation	44 (25.3 %)

Abbreviations: PCN-Plasma cell neoplasms, MM- Multiple Myeloma, NHL-Non-Hodgkin's lymphoma, HL- Hodgkin's lymphoma ISS-International Staging System, R-ISS-Revised International Staging System, G-CSF-Granulocyte Colony Stimulating Factor, P-plerixafor, CAD-Cyclophosphamide, Adriamycin, dexamethasone, N/A- not available.

### PBSC count and mobilization outcomes

On the first day of enumeration, the median number of CD34^+^ cells was 25.5 cells/µL (range 0–248). The median number of circulating CD34^+^ cells was 65 cells/µL (range 5–248) in the group of patients that did not require plerixafor, while for the patients that needed plerixafor the median was 14 cells/µL (range 0–56). In accordance with the GITMO definition, 72 patients were proven poor mobilizers (PM) with a count ≤ 20 circulating CD34^+^ cells/µL of PB. Patients mobilized after CAD chemotherapy had higher PBSC counts than PCN patients mobilized using only cytokines, *p*< 0.001. Lymphoma patients had lower PBSC counts after disease specific chemotherapy mobilization with the median being 4 cells/µL (range 0–198) compared to 14.5 cells/µL (range 0–89) in patients mobilized only using G-CSF, *p* = 0.046. Plerixafor led to a 312 % increase in the median number of circulating CD34^+^cells 6 h after administration. The median value of CD34^+^ cells on the apheresis day was 61 cells/µL (range 6–594). Higher peak circulating CD34^+^ values were achieved after chemotherapy-based mobilization comparing to steady state mobilization in both PCN and lymphoma patients, *p*< 0.001 and *p* = 0.004, respectively. Univariate regression analysis showed a high correlation between CD34^+^ counts and apheresis yields, *p*< 0.001. Out of the 173 patients, the majority of patients, 101 (58.4 %) received at least one dose of plerixafor, 24 patients (13.9 %) received a total of 2 plerixafor doses. In the group of proven PM 66 patients received plerixafor (91.6 %) The need for plerixafor administration was diagnosis dependent, it was required in half of all PCN patients compared to 75 % lymphoma patients *p* = 0.008. Out of the 173 patients undergoing mobilization, only one patient (0.58 %) did not proceed to stem cell apheresis. On the fifth day of G-CSF administration, 48 patients (27.7 %) achieved adequate CD34^+^ counts and proceeded to apheresis. An additional 24 patients (13.9 %) were able to proceed after dose escalation of G-CSF without the need for plerixafor. Seven patients (4 %) were unable to achieve a collection total of ≥ 2 × 10^6^ CD34^+^ cells/kg bm within two consecutive days of apheresis even with the use of plerixafor. Mobilization and apheresis outcomes are summarized in Table 2. PCN patients mobilized after CAD chemotherapy required less plerixafor administration than patients mobilized in only with G-CSF, *p* = 0.01, while there was no difference in the need for plerixafor when comparing lymphoma patients mobilized from salvage regimens and those mobilized only using cytokines *p* = 0.46. The median collected dose in the patient population was 7.8 × 10^6^ (range 1.06–26.14) CD34^+^ cells/kg bm (Table 3). The majority of patients (67.6 %) achieved optimal cell collection doses, 48 patients (27.7 %) patients had adequate and 7 (4 %) patients had inadequate cell doses. Almost all patients (90 %) achieved ≥ 2 × 10^6^ CD34^+^ cells/kg bm after one apheresis session with the median after first apheresis being 7.72×10^6^ (range 0.86–26.16) CD34^+^ cells/kg bm.

**Table 2:** Mobilization and apheresis outcomes.

	First enumeration CD34^+^ cells/µL (range)	Count on apheresis CD34^+^ cells/µL (range)	Total apheresis harvest 10^6^ x CD34^+^ cells /kg bm (range)
PCN			

Steady state G-CSF Steady state G-CSF+*P* P value CAD G-CSF CAD G-CSF+*P* P value All steady state ALL CAD P value	54 (34–150)	54 (34–150)	5.05 (3.24–14.3)
	17 (1–56)	49 (14–134)	6 (1.36–12.8)
	< 0.001	0.024	0.49
	77.5 (19–248)	79.5 (26–594)	11.4 (4.2–26.14)
	21 (2–47)	116 (29–227)	12.9 (2.84–26.02)
	< 0.001	0.55	0.60
	26 (1–150)	54 (14–150)	5.7 (1.36–14.3)
	47 (2–248)	88 (26–594)	11.3 (2.84–26.14)
	0.002	< 0.001	< 0.001

Lymphoma			

Steady state G-CSF Steady state G-CSF+*P* P value Salvage G-CSF Salvage G-CSF+*P* P value All steady state All salvage P value	41 (7–89)	52 (34–89)	5.7 (3.5–7.3)
	8 (0–31)	26 (6–61)	3.8 (1.06–9.61)
	0.015	0.035	0.404
	39.5 (5–198)	68 (24–396)	9.48 (2.36–18.05)
	4 (0–31)	54 (19–146)	7.3 (1,74–17.3)
	< 0.001	0.389	0.167
	14.5 (0–89)	41 (6–89)	4.09 (1.06–9,61)
	4 (0–198)	60 (19–396)	7.5 (1.74–18.05)
	0.046	0.004	< 0.001

Abbreviations: PCN-Plasma cell neoplasms, MM- Multiple Myeloma, NHL-Non-Hodgkin's lymphoma, HL-Hodgkin's lymphoma, G-CSF-Granulocyte Colony Stimulating Factor, P-plerixafor, CAD-Cyclophosphamide, Adriamycin, dexamethasone.

**Table 3:** Mobilization and apheresis outcomes between groups.

Variable	G-CSF (A)	G-CSF+*P* (B)	All patients	Statistical significance, (p value) between groups A & B
CD34^+^ cells/µL on first enumeration (range)	65 (5–248))	14 (0–56)	25.5 (0–248)	< 0.001
CD34^+^ cells/µL on first apheresis day (range)	73.5 (24–594)	54.5 (6–227)	61 (6–594)	< 0.001
CD34^+^ cells collected on the first apheresis day /kg bm (range)	9.11 (2.36–26.14)	6.5 (0.85–20.02)	7.72 (0.86–26.14)	0.002
Total CD34^+^/kg bm harvested (range)	9.19 (2.37–26.14)	7.27 (1.06–20.02)	7.8 (1.06–26.14)	0.002
Optimal > 5 (> 10 for tandem) x10^6^ CD34^+^ cells /kg bm	54 (31.2 %)	63 (36.4 %)	117 (67.8 %)	0.08
Adequate 2–5 (8–10 for tandem) x10^6^ CD34^+^ cells /kg bm	18 (10.4 %)	30 (17.3 %)	48 (27.7 %)	0.588
Inadequate < 2 × 10^6^ CD34^+^ cells/kg bm	0 (0 %)	7 (4.0 %)	7 (4.0 %)	0.042
Number of multiple apheresis sessions	3 (1.7 %)	14 (8.1 %)	17 (9.8 %)	0.039

### Hematopoietic recovery and patient outcomes after transplantation

A total of 172 patients underwent ASCT. Of the 111 MM patients, tandem transplantation was performed in 44 (39 %). In the first transplantation, all patients achieved engraftment but 4 patients died, 3 from infection and one due to a fatal gastrointestinal bleed, while in the second transplantation two patients died in the pre-engraftment phase due to infection related complications. In total, six patients died after ASCT (3.48 %). The median time needed for neutrophil engraftment was 11 days (range 9–18) and 13 days (range 10–26) for platelets. Time to engraftment was longer in the second tandem transplantation for both neutrophils and platelets, 11 (range 9–18) and 13 (range 10–26) after the first transplantation compared to 12 (range 10–25) and 13 (range 11–31) in the second transplantation *p*< 0.001. Time to platelet engraftment was longer in the group of patients that received plerixafor *p* = 0.029, no impact on neutrophil engraftment was observed, *p* = 0.219. Engraftment kinetics are shown in Table 4. Patients receiving optimal cell doses had a faster hematopoietic recovery when comparing them to those receiving suboptimal HSC doses, *p*< 0.001. All patients had stable long term hematopoietic reconstitution at the time of the first follow up. Patients with the diagnosis of PCN achieved VGPR or better (VGPR + CR) in 85 patients (69.1 %) after standard induction, following ASCT 101 (82.1 %) patients achieved VGPR + CR, *p*< 0.001. In the group of lymphoma patients, 32 (64 %) achieved PR and 9 (18 %) achieved CR after following salvage chemotherapy. After ASCT 22 (44 %) patients were in CR and 14 (28 %) were in PR, *p* = 0.248.

**Table 4:** Characteristics of engraftment.

Days to engraftment (range)	All patients	G-CSF	G-CSF + P
Neutrophils in single transplant or on 1st tandem transplantation	11 (9–18)	11 (9–14)	11 (9–18)
Neutrophils in 2nd tandem transplantation	12 (10–25)	12 (10–16)	13 (10–25)
Platelets in single transplant or on 1st tandem transplantation	13 (10–26)	13 (10–21)	13 (10–26)
Platelets in 2nd tandem transplantation	13 (11–31)	13 (11–26)	13 (11–31)

## Discussion

Mobilization failure or use of inadequate stem cell doses can have major detrimental effects on patients undergoing ASCT [12], [13], [14]. The use of plerixafor in the first mobilization attempt either after steady state mobilization or after chemotherapy-based mobilization reduces the need for multiple apheresis sessions and mobilization failure rates thus reducing overall mobilization costs. The optimization of apheresis with adequate use of plerixafor, even considering its high price, can ameliorate the cost of prolonged hospitalization and high utilization of healthcare resources. Finally, this approach improves patient comfort [33], [34], [35]. Optimization of mobilization process also provides an adequate HSC number in the harvest after apheresis. Therefore, utilization of a combined approach in accordance with the EBMT guidelines and especially *just in time plerixafor* results in reduced failure rates and represents the ultimate goal [5,36,37].

Numerous studies, including our own, have shown that low circulating CD34^+^ cell numbers after mobilization are a strong predictor of inadequate cell harvests [38], [39], [40]. After 4 days of G-CSF administration, the median number of CD34^+^cells was 65 cells/µL of PB in the G-CSF only group, while in the group of patients requiring plerixafor the median number was only 14 cells/µL of PB. A single dose of plerixafor successfully overcame an inadequate response to G-CSF by increasing the median number of PBSC by 312 % after 6 h. Similar results were reported by Stiff P et al. during phase II trials and Di Persio et al. during phase III trials [41,42].

Mobilization protocol CAD led to higher values of PBSC, both on the first day of enumeration and on the first apheresis day, thus resulting in a lower need for plerixafor. This beneficial effect of chemotherapy-mobilization was not observed in lymphoma patients. We can speculate that this is due to the majority of lymphoma patients in our study being heavily pretreated before mobilization contrary to PCN patients who received only one line of therapy in the majority of cases [22]. Another compounding factor for the responsiveness of PCN patients could be the scarce use of novel drugs for induction like lenalidomide or daratumumab, which are known to impact stem cell mobilization [43,44].

Selected chemotherapy-based mobilization and less stringent adherence to plerixafor use guidelines led to a failure rate of only 4 % all with the absolute majority of patients reaching sufficient yields after one apheresis session. In this study collection of > 2 × 10^6^ CD34^+^/kg bm was achieved in 96 % of all patients, and 73.8 % achieved yields > 5 × 10^6^ CD34^+^ cells/kg bm. One apheresis session was enough to successfully collect target amounts of SC in 155 patients (90.1 %) while 17 patients required an additional apheresis on the day after. No patients required more than 2 apheresis sessions. These results are comparable with the use of upfront plerixafor both in failure rates and in total number of cells harvested [45,46]. This approach has proved effective in our institution reducing the number of apheresis sessions, duration of hospitalization length and most importantly it allowed patients to successfully and promptly proceed to ASCT without delays.

In accordance with the American Society for Blood and Bone Marrow Transplantation (ASBMT) recommendations for minimal cell dose infused, all of our patients that underwent apheresis were transplanted [27]. All patients successfully engrafted in the first transplantation, but 4 patients died, 3 due to infection, 1 due to a lower gastrointestinal bleed. In the second ASCT two patients died before engraftment due to infection. Patients receiving plerixafor had marginally longer times to platelet engraftment. This is most likely due to lower cell doses infused, but it can also be potentially attributed to the higher cumulative doses of G-CSF used and its impact on CXCR4 expression on PBSC [47]. Time to engraftment was comparable to other studies of this nature, with Serin et al. also reporting a longer time to platelet engraftment in plerixafor exposed patients [48], [49], [50]. Only one patient did not achieve engraftment after 30 days even with the infused dose being adequate. The graft durability was excellent and all patients that survived transplantation had adequate long term hematopoietic reconstitution at the time of first follow-up, which indicates that plerixafor can mobilize high quantity of quality cells needed for hematopoietic reconstitution.

The limitations of our study are its retrospective nature, lack of uniformity of cell dose required for ASCT, and lack of stringent criteria for plerixafor use.

## Conclusion

We conclude that properly selected plerixafor administration reduces the incidence of "mobilization-related-failure" and assure a high-level cell dose for SC transplants, with following superior "therapeutic-potential" and safety profile. A mobilization approach that includes "*just-in-time*" plerixafor administration, also leads to a reduction of hospitalization days and healthcare resource utilization. For definitive conclusions further controlled and larger clinical trials concerning correlation of circulating and harvested CD34^+^ cell count/yield, with hematopoietic reconstitution are required.

## CRediT authorship contribution statement

**Milena Todorović Balint:** Conceptualization, Methodology, Writing – review & editing. **Nikola Lemajić:** Data curation, Writing – original draft. **Vladimir Jurišić:** Methodology, Supervision. **Sofija Pantelić:** Data curation, Writing – original draft. **Dejana Stanisavljević:** Methodology, Validation. **Nada Kraguljac Kurtović:** Supervision. **Bela Balint:** Writing – review & editing, Conceptualization, Supervision.

## Declaration of Competing Interest

None.
